# Health Alliance for Prudent Prescribing, Yield and Use of Antimicrobial Drugs in the Treatment of Respiratory Tract Infections (HAPPY AUDIT)

**DOI:** 10.1186/1471-2296-11-29

**Published:** 2010-04-23

**Authors:** Lars Bjerrum, Anders Munck, Bente Gahrn-Hansen, Malene Plejdrup Hansen, Dorte Jarboel, Carl Llor, Josep Maria Cots, Silvia Hernández, Beatriz González López-Valcárcel, Antoñia Pérez, Lidia Caballero, Walter von der Heyde, Ruta Radzeviviene, Arnoldas Jurgutis, Anatoliy Reutskiy, Elena Egorova, Eva Lena Strandberg, Ingvar Ovhed, Sigvard Molstad, Robert vander Stichele, Ria Benko, Vera Vlahovic-Palcevski, Christos Lionis, Marit Rønning

**Affiliations:** 1 Research Unit of General Practice, University of Southern Denmark, Odense, Denmark; 2 Spanish Society of Family Medicine, Barcelona, Spain; 3 University of Las Palmas de Gran Canarias, Las Palmas, Spain; 4 Misiones Association of General Family Medicine, Posadas, Argentina; 5 Public Health Department, Klaipeda, Lithuania; 6 Association of Family Doctors, Kaliningrad, Russia; 7 Department of Clinical Sciences, Lund University, Malmoe, Sweden; 8 European Drug Utilisation Research Group, Ghent, Belgium; 9 World Organisation of Family Doctors (WONCA), Lubljana, Slovenia; 10 World Health Organisation, Collaborating Centre for Drug Statistics Methodology, Oslo, Norway; 11 Current address: The Research Unit for General Practice and Section of General Practice, Department of Public Health, University of Copenhagen, Copenhagen, Denmark

## Abstract

**Background:**

Excessive and inappropriate use of antibiotics is considered to be the most important reason for development of bacterial resistance to antibiotics. As antibiotic resistance may spread across borders, high prevalence countries may serve as a source of bacterial resistance for countries with a low prevalence. Therefore, bacterial resistance is an important issue with a potential serious impact on all countries.

The majority of respiratory tract infections (RTIs) are treated in general practice. Most infections are caused by virus and antibiotics are therefore unlikely to have any clinical benefit. Several intervention initiatives have been taken to reduce the inappropriate use of antibiotics in primary health care, but the effectiveness of these interventions is only modest. Only few studies have been designed to determine the effectiveness of multifaceted strategies in countries with different practice setting. The aim of this study is to evaluate the impact of a multifaceted intervention targeting general practitioners (GPs) and patients in six countries with different prevalence of antibiotic resistance: Two Nordic countries (Denmark and Sweden), two Baltic Countries (Lithuania and Kaliningrad-Russia) and two Hispano-American countries (Spain and Argentina).

**Methods/Design:**

HAPPY AUDIT was initiated in 2008 and the project is still ongoing. The project includes 15 partners from 9 countries. GPs participating in HAPPY AUDIT will be audited by the Audit Project Odense (APO) method. The APO method will be used at a multinational level involving GPs from six countries with different cultural background and different organisation of primary health care. Research on the effect of the intervention will be performed by analysing audit registrations carried out before and after the intervention. The intervention includes training courses on management of RTIs, dissemination of clinical guidelines with recommendations for diagnosis and treatment, posters for the waiting room, brochures to patients and implementation of point of care tests (Strep A and CRP) to be used in the GPs'surgeries.

To ensure public awareness of the risk of resistant bacteria, media campaigns targeting both professionals and the public will be developed and the results will be published and widely disseminated at a Working Conference hosted by the World Association of Family Doctors (WONCA-Europe) at the end of the project period.

**Discussion:**

HAPPY AUDIT is an EU-financed project with the aim of contributing to the battle against antibiotic resistance through quality improvement of GPs' diagnosis and treatment of RTIs through development of intervention programmes targeting GPs, parents of young children and healthy adults. It is hypothesized that the use of multifaceted strategies combining active intervention by GPs will be effective in reducing prescribing of unnecessary antibiotics for RTIs and improving the use of appropriate antibiotics in suspected bacterial infections.

## Background

Excessive and inappropriate use of antibiotics is considered to be the most important reason for development of bacterial resistance to antibiotics [1-3]. Countries with a high use of antibiotics like the Southern European countries have a high rate of resistance, while the Nordic countries with a low use have a low resistance rate [4]. As antibiotic resistance may spread across borders, high prevalence countries may serve as a source of bacterial resistance for countries with a low prevalence. Therefore, bacterial resistance is an important issue with a potentially serious impact on all countries.

Infections caused by resistant bacteria lead to an increased mortality, prolonged hospital stay and increased costs [5,6]. History has told us that this problem will not be solved by the provision of more potent antibiotics by the pharmaceutical industry - quite the contrary. The control of antibiotic resistance should be solved by other initiatives. A cornerstone of efforts to control antibiotic resistance is to improve the quality of antibiotic prescribing in primary health care, as more than 90% of antibiotics are prescribed by GPs. The majority of antibiotics prescribed in general practice are for respiratory tract infections (RTIs) which constitute approximately 70% of all infections treated in family practice [7,8]. The majority of RTIs (90%) are caused by virus and in these cases antibiotics are unlikely to have any clinical benefit for the patient. Most RTIs are harmless and self-limiting and nearly all patients recover without any specific treatment. Antibiotic treatment may thus be superfluous, and in some cases it may be directly harmful due to adverse effects. Even if the aetiology is bacterial, antibiotics modify RTIs only slightly, particular in patients with upper RTIs [9,10].

### Previous research

It has been clearly documented that the prevalence of resistant strains is correlated with the consumption of antibiotics, and studies comparing bacterial resistance in various European countries have shown striking differences in the consumption of antibiotics [4,11]. Until recently, the rates of antibiotic resistance in the northern European countries have remained low. However, the rates of resistance in the southern European countries are reaching alarming levels.

Studies of the management of RTIs show that a considerable number of antibiotic prescriptions are neither necessary nor appropriate [12,13]. Studies from general practice have shown that more than half of all patients with colds and respiratory tract infections (RTIs) and about nine out of ten patients with suspected bronchitis are treated with antibiotics. The different antibiotic prescribing rates between countries cannot be explained by different frequency or patterns of RTIs. More likely, it is due to discrepancies in national recommendations, different health care systems, different treatment traditions, different culture, different patient expectations or different impact of marketing by pharmacies and pharmaceutical companies.

Several initiatives have been taken to reduce the inappropriate use of antibiotics in primary health care. However, most interventions have not achieved impressive results [14]. Printed educational material and didactic lectures only have little influence on prescribing habits. According to a review from the *Cochrane Library*, multifaceted interventions seem to be more effective than singular interventions [14]. However, only a few multifaceted interventions targeting treatment of RTIs have been performed.

Most studies investigating consumption of antibiotics in different countries have been based on aggregated data from prescription databases or from wholesale figures. Such data do generally not contain information about the indication for prescription. As the vast majority of antibiotic prescriptions in general practice are issued for RTIs, studies comparing GPs' management of different RTIs are suitable for exploring the consumption of antibiotics in more detail. Particularly, information about the background for prescribing in countries with different prevalence of antibiotic resistance is needed.

Only few studies have been designed to evaluate and compare the effect of multifaceted interventions in countries with different practice settings. Furthermore, we need more knowledge about the effect of combining interventions. Particularly, we need information about factors that may influence the effect of interventions targeting doctors and patients from different practice settings.

The aim of this study is to evaluate the impact of a multifaceted intervention including an active intervention by GPs on their antibiotic prescribing in six countries with different prevalence of antibiotic resistance: Two Nordic countries (Denmark and Sweden), two Baltic Countries (Lithuania and Kaliningrad-Russia) and two Hispano-American countries (Spain and Argentina).

## Methods/Design

HAPPY AUDIT was initiated in 2008 and the project is still ongoing; it is planned to be finalized by the end of 2010. The project includes 15 partners from 9 countries, as indicated in Table 1.

**Table 1:** Partners in HAPPY AUDIT

Participant organisation name
Research Unit of General Practice, University of Southern Denmark
General Practice Consultants, Odense, Denmark
Ministry of the Interior and Health, Denmark
Department of Clinical Sciences, Lund University, Sweden
National Board of Health and Welfare, Sweden
Family Doctor (FD) Centre, Lithuania
State Patient Fund; Lithuania
Association of Family Doctors, Kaliningrad, Russia
Spanish Society of Family Medicine, Spain
University of Las Palmas de Gran Canarias, Spain
Consejería de Sanidad del Gobierno de Canarias, Spain
Misiones Association of General Family Medicine, Posadas, Argentina
World Health Organisation, Collaborating Centre for Drug Statistics Methodology, Oslo, Norway
World Organisation of Family Doctors (WONCA), Slovenia
European Drug Utilisation Research Group, Belgium

The proposed method for auditing GPs participating in HAPPY AUDIT is called Audit Project Odense (APO) [15]. APO has been developed and successfully tested among different groups of GPs in the Nordic countries [16].

In this project APO will be used at a multinational level involving GPs from six countries with different cultural background and different organisation of primary health care. Research on the effect will be performed by analysing audit registrations carried out before and after the intervention period. Recommendations for health policy at political level will be prepared. The results will be widely disseminated at a Working Conference at the end of the project period.

**Figure 1:**
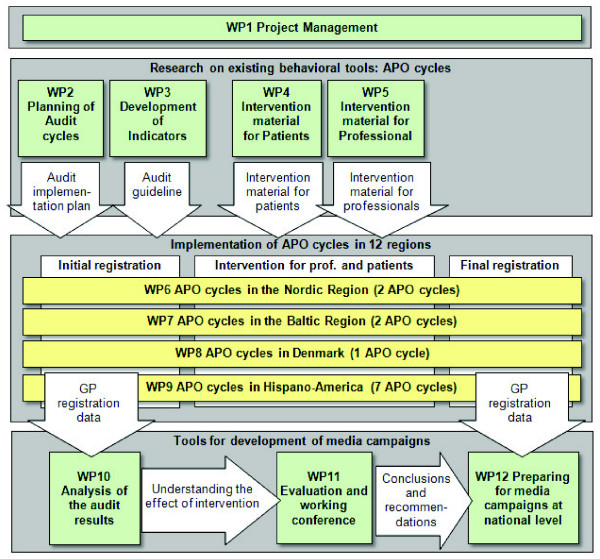
**HAPPY AUDIT structure**.

HAPPY AUDIT is structured into 12 work packages (WPs) and organized in 3 groups as indicated in Figure 1.

1. Research on existing behavioural tools: APO cycles

• Planning of Audit cycles (WP2)

• Development of quality indicators (WP3)

• Preparation of intervention materials for patients (WP4) and professionals (WP5)

2. Implementation of APO cycles (WP6, WP7, WP8 and WP9)

• Initial registration of patients with RTIs (before intervention)

• Intervention activities targeted professionals and patients

• Final registration of patients with RTIs (after intervention)

3. Developing tools for development, implementation and evaluation of media campaigns

• Analyzing the effect of the intervention (WP10)

• Working Conference with presentation of recommendations from the study (WP11)

• Preparing for media campaigns at national level (WP12)

After completion of WP2-WP5, the initial APO registration will take place. The audit cycles are performed in six countries with contrasting prevalence of antibiotic resistance: two countries with low consumption of antibiotics and low resistance rates (Sweden and Denmark), two countries with high consumption of antibiotics and high resistance rates (Spain and Argentina), and two countries with an increasing consumption of antibiotics and increasing resistance rates (Lithuania and Russia).

**Figure 2:**
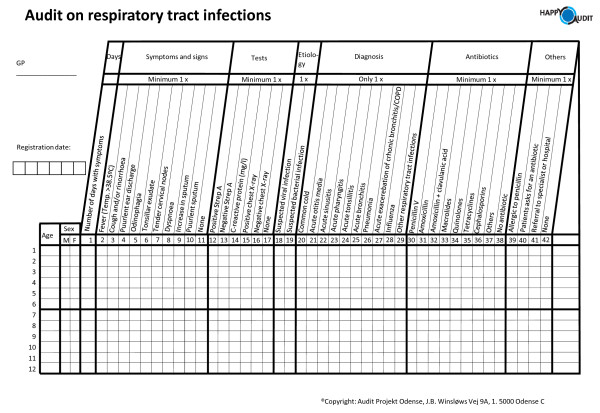
**The HAPPY AUDIT registration sheet**.

According to the APO method, patients will be registered using a prospective self-registry methodology based on a simple chart completed by the GP during the consultation (Figure 2).

A report will be prepared and subsequently discussed at the first follow-up meting during the intervention phase. The participants will discuss their own performance, identify quality problems and consider possible barriers and solutions to elimination of the quality problems.

Each GP will register patients during a 3-week period before and after the intervention.

The minimum number of GPs per audit cycle has been estimated based on the following assumptions: Each GP will register about 25 consultations during the 3 weeks of audit registration. A decrease of 10% in antibiotic prescribing can be expected after the intervention (from about 40% before to about 30% after). The within-practice correlation coefficient is 0.1.

If the comparison is to be performed at the 5% level of significance (two-sided) with 80% power, then the number of GPs to be included should be 48 per APO cycle.

### Intervention

**Figure 3:**
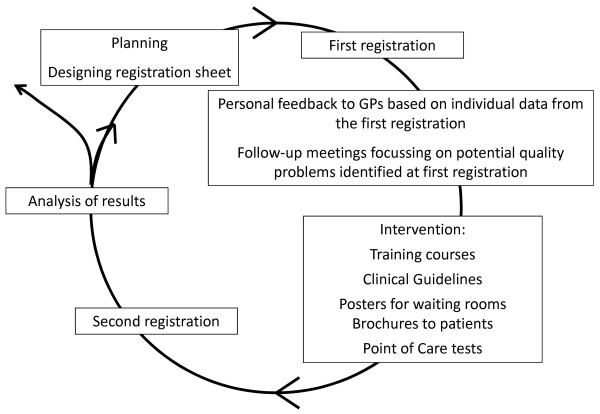
**The Audit project Odense method**.

The follow-up intervention activities will comprise workshops, clinical skills courses, reminders and clinical training, as well as interdisciplinary training courses (Figure 3). The intervention also includes introduction of diagnostic classification of patients with RTIs by using microbiological laboratory point of care (POC) tests: Streptococcus Antigen test (Strep A) and C-reactive Protein test (CRP).

After the first registration, the GPs will receive personal feedback based on individual data on results from the first registration. Furthermore, they will receive feedback from the group of GPs in their country based on aggregated data on results from the first registration. The results will be discussed at follow-up meetings, and potential quality problems identified and intervention activities initiated. The intervention will start after the first registration period and it will include the following activities:

• Training course on appropriate use of antibiotics for RTIs

• Clinical guidelines including recommendations for diagnosis and treatment of RTIs

• Posters for doctors' waiting rooms, focusing on the appropriate use of antibiotics, and targeting all patients visiting practice

• Brochures and handouts to patients about prudent use of antibiotics

• Point of care (POC) rapid tests: Strep A and CRP

• Training in use and interpretation of results from POC rapid tests

After the intervention period, the GPs will perform the second registration during a 3-week period one year later.

In order to evaluate the influence of specific elements of the intervention (e.g. introduction of POC tests) GPs will be exposed to different types and combinations of intervention activities during the study. The majority of GPs will be exposed to prescription feedback, guidelines, courses and POC tests. A group of GPs will only be offered prescription feedback and guidelines, but no POC test. Another group of GPs will not be exposed to any type of intervention activities, except performance of an audit cycle (control group).

### Analysis

The statistical analysis will provide an understanding of the effect of intervention. GPs' prescribing of antibiotics for patients with RTIs will be compared in countries with high prevalence of antibiotic resistance among respiratory pathogens (Hispano-America and the Baltic Countries) and countries with low prevalence of antibiotic resistance (Denmark, Sweden).

The ecological fallacy introduced by using aggregated data may mislead strategies to combat antibiotic resistance. Analyses of data of individual patients are therefore essential to direct policies in strategies to combat antibiotic resistance. The method proposed for the analysis is a multilevel model to determine how the clinical prescription practice has changed after the intervention. The sample selection of physicians, the inclusion criteria and recruitment mechanism will be designed to get as good statistical information as possible with the data coming from a natural hierarchy of units grouped at different levels:

• The type of RTI in patients

• The GPs

• The organisation of primary health care in different regions or countries

The aim of multilevel models in HAPPY AUDIT is to analyse the results of the audit (comparing before and after the audit) with regard to a number of dependent variables: appropriateness of antibiotic prescriptions (multilevel discrete choice models); number and rate of antibiotic prescriptions, expenditure and others. These models allow us to test if there are environmental and organisational factors influencing the effectiveness of the audit (Are there any significant differences among regions? Are there some characteristics of the GPs influencing the audit results?)

The data will be analyzed by the statistical program Stata, version 11. The chi-squared, Student's t tests, and analysis of variance will be used to compare proportions and means, respectively. Multilevel logistic regression analyses will be performed to evaluate the effect of the intervention at different levels. Statistical significance will be considered with a p value < 0.05.

### Limitations of the study

GPs participate on a voluntary basis and probably their prescribing habits may not represent the average use of antibiotics in their country [17]. GPs participating in audits may be more interested in quality development and research than GPs in general. Furthermore, they are willing to dedicate time to complete audit reports without economic incentives.

The amount of time involved in a quality improvement project could be considered to be a prominent barrier to participation, as GPs may find it difficult to find the time in their daily work. However, participation in an APO registration is not very time-consuming. Each registration takes less than 2 minutes. But the GPs need to set aside time for the subsequent courses or other activities planned in the intervention.

Another limitation which should be taken into account is the fact that performing an audit may in itself influence the prescribing habits. However, studies have shown that the reliability of the *Audit Project Odense *methodology applied in different countries is high and findings are correlated with the real prescription in practice [15].

From a theoretical point of view, the decision to treat should be taken after a diagnosis has been established. In general practice, however, the diagnostic procedures and the decision to treat are intricately linked. The GP may decide whether or not to prescribe an antibiotic at the same time, or even before, he classifies a specific diagnosis to the patient. After making the decision to prescribe the GP may thus adjust the diagnosis to fit the decision about treatment. This may lead to a diagnostic misclassification bias. However, this potential bias will affect the validity of the diagnosis both before and after the intervention and it only has a small likelihood of influencing the effect of the intervention.

Due to the limited time allocated for the registration process in practice only the typical signs and symptoms for RTIs according to the medical literature will be recorded. This may lead to some limitations. Non-biomedical factors that may represent very powerful predictors of antibiotic prescription will not be taken into account in this study.

The before-after design used in this study suffers from some limitations due to the fact that changes in antibiotic prescribing could be due to other factors than the intervention performed by the investigators.

### Ethical considerations

All patient registration data will be treated confidentially according to the law on protection of sensitive data. The data will be organised in a database and made available for analysis in this project and for research in future projects. The project will be conducted in accordance with the EU Directive of good clinical practice (EU Directive 2001/20/EC).

During the APO cycle, the participating GPs will be exposed to different interventions, but patients will not undergo any intervention. Therefore, patients will not be asked for informed consent. Patients will be informed about the objective of the project and they will be told that specific clinical information related to the consultation will be entered into a multinational database. Appropriate protection of patient information is paramount and all data from the project will be processed according to the European Community Data Protection Directive (EU-Directive 95/46/EC) on the protection of individuals with regard to the processing of personal data.

Patients will be registered by age and sex only. No electronic patient identifier (such as civil registration number) will be used, and there will be no information that can be used to identify individual registry patients by personal analysis of the data. The identity of the participating GPs will be considered to be confidential information. All personnel with access to data containing personal identifiers will sign a pledge to maintain the confidentiality of study subjects. All data management and statistical analysis programs used in the analyses will be documented.

Approval has been obtained from the Ethical Committees of Investigation in Primary Care in the six participating countries.

## Discussion

Despite the exaggerated use of antibiotics and the growing development of bacterial resistance, only few initiatives have been taken to reduce the inappropriate use of antibiotics. The aim of the HAPPY AUDIT study is to evaluate the impact of a multifaceted intervention programme focusing on appropriate treatment of RTIs and targeting general practitioners and patients in general practice.

The study takes place in six countries with contrasting prevalence of antibiotic resistance. The intervention will combine a number of intervention initiatives, including feedback to GPs, training courses, follow-up meetings, guidelines, posters for waiting rooms, brochures to the patients, and implementation of POC tests (Strep A and CRP) in practice.

The APO cycles in this study will be performed in a natural practice setting, and patients will not be informed about the project prior to the consultations. GPs participating in the audit will not get allocated extra time for consultations, and they will not be able to make considerable changes in their practice activities during the 3 weeks of registration. Thus, they will see the same patients as if they were not participating in the audit.

Therefore, it is most likely that the results from the project can be extrapolated to other areas and used to influence other practices with similar settings.

## Competing interests

The authors declare that they have no competing interests.

## Authors' contributions

LB and CL drafted the manuscript. All the authors participated in the design of the study and offered critical revisions. All authors read and approved the manuscript.

## Pre-publication history

The pre-publication history for this paper can be accessed here:

http://www.biomedcentral.com/1471-2296/11/29/prepub
